# Risk factors of dropout from institutional delivery among HIV positive antenatal care booked mothers within one year postpartum in Ethiopia: a case–control study

**DOI:** 10.1186/s13690-022-00819-0

**Published:** 2022-02-25

**Authors:** Muhabaw Shumye Mihret, Zelalem Nigussie Azene, Azmeraw Ambachew Kebede, Banchigizie Adane Mengistu, Getachew Azeze Eriku, Mengstu Melkamu Asaye, Wagaye Fentahun Chanie, Birhan Tsegaw Taye

**Affiliations:** 1 Department of Clinical Midwifery, School of Midwifery, College of Medicine and Health Sciences, University of Gondar, Gondar, Ethiopia; 2 Department of Women’s and Family Health, School of Midwifery, College of Medicine and Health Sciences, University of Gondar, Gondar, Ethiopia; 3 Department of Midwifery, Teda Health Science College, Amhara Regional Health Bureau, Gondar, Ethiopia; 4 Department of Physiotherapy, College of Medicine and Health Sciences, University of Gondar, Gondar, Ethiopia; 5 College of Medicine and Health Sciences, UNFPA Supported Maternal Health Project Coordinator, University of Gondar, Gondar, Ethiopia; 6 School of Nursing and Midwifery, Asrat Weldeyes Health Science Campus, Debre Berhan University, Debre Birhan, Ethiopia

**Keywords:** Case–Control, Continuum of Care, Determinants, Institutional delivery, HIV-positive

## Abstract

**Background:**

The uptake of maternal healthcare services remains suboptimal in Ethiopia. Significant proportions of antenatal care attendees give birth at home in the context of HIV. However, in Ethiopia, evidence is scarce on the predictors of dropout from maternity continuum of care among HIV-positive mothers. Therefore, this study aimed to supply valuable information on risk factors regarding dropout of HIV-positive mothers for institutional delivery services in northwest Ethiopia.

**Methods:**

A multicenter case–control study was conducted at governmental health facilities in Gondar City from May one to June 30/2018. A total of 222 HIV-positive women were included in the study. Data were collected using structured questionnaires and checklists through face-to-face interview and chart review; entered into EPI INFO version seven, and then exported to SPSS version 25. Both descriptive and analytical procedures were performed. Binary logistic regression analysis was undertaken. A significant association was declared based on the adjusted odds ratio (AOR) with its 95% CI and p-value of ≤ 0.05.

**Results:**

This study illustrates that maternal age of ≥ 35 years (AOR = 2.37; 95%CI: 1.13,5.13), unmarried marital relation (AOR = 3.28; 95%CI: 1.51, 7.13), unemployed spousal occupation (AOR = 3.91; 95%CI: 1.54, 9.91), family monthly income of ≤ 36 US dollar (AOR = 4.87; 95%CI: 2.08, 11.42) and no obstetric complication in the index pregnancy (AOR = 13.89; 95%CI: 2.73, 27.71) were positively associated with dropout from institutional delivery among HIV positive antenatal care booked mothers.

**Conclusion:**

In this study, the risk factors of dropout from institutional delivery in the context of HIV-positive women were connected to social determinants of health such as advanced maternal age, unmarried marital status, unemployed husband occupation, and low family income. Therefore, interacting with the health system by focusing on these women in lower socio-economic strata and unmarried HIV-positive ANC attendees, and increasing access to information on obstetric complications during the antenatal care visit would retain clients in the continuum of maternity services.

**Supplementary Information:**

The online version contains supplementary material available at 10.1186/s13690-022-00819-0.

## Background

About 36.9 million people are living with the Human Immune Deficiency Virus (HIV) in the world. Of these, around 1.5 million people are pregnant women and the majority of those pregnant women are found in Sub-Saharan Africa (SSA) which accounts for 50 to 90% of all the global HIV infections [1–3]. Efforts are made to combat this problem. For example, the newly accepted global agenda between 2016 and 2030 as a part of the sustainable development goal (SDG) is directed to ensure good health and wellbeing of women. This goal is specifically targeted to decrease the maternal mortality ratio (MMR) to 70 per 100, 000 live births by addressing barriers to all maternal health care services utilization for every single woman as the highest priority [4, 5]. However, women in underdeveloped countries including Ethiopia, dropout before finishing the full course of maternity continuum of care (MCOC) remains a bottleneck to achieving so.

Dropout from MCOC (i.e., inabilities to finish antenatal care (ANC) follow-up, failure to give birth in the health facilities, and failure to get postnatal checkup) is unsafe for both the women and the newborn [6]. This failure to access skilled birth attendants (SBA) could result in compromised health and wellbeing of both the mother and the newborn [7]. Moreover, MCOC is a great prospect in the struggle to decrease maternal and perinatal mortality, giving more advantages to HIV-positive pregnant women [8].

Though the effects of HIV infection on maternal morbidity, mortality, and vertical transmission to her baby are well-recognized, the association between maternal HIV infection and adverse pregnancy outcomes is still controversial [9]. But, the burden of those adverse pregnancy outcomes is believed to be higher if the women had poor adherence to MCOC and dropout from institutional delivery as it could embarrass the prevention of mother to child transmission (PMTCT) of HIV.

Hence, determinants of home delivery among HIV-positive women after booking for ANC need to be investigated so that stakeholders can apply evidence-based interventions [10]. Existing evidence shows that women with high insight of HIV-related stigma, discrimination, and unreceptive attitude of health workers are highly likely to dropout from MCOC and they are not eager to deliver their baby in a health institution by SBA [11, 12]. This knowing avoidance due to fear of stigma could result in obstetrics complications, mother-to-child transmission (MTCT) of the virus, and preventable maternal mortality [13–15].

In Ethiopia, trends of reproductive health indicators from 2000–2014 and the Ethiopian demographic health survey (EDHS) 2016 report revealed that the dropout rate from MCOC is still high despite the fact there is substantial maternal health care services utilization [11, 16]. According to the Ethiopian mini demographic and health survey (EMDHS) 2019 report, about 74% of pregnant women received ANC at least once. However, only 48% of them gave birth at a health facility [17]. This implies that a significant proportion of the ANC booked women are dropped out of the MCOC due to several reasons. Even though studies were done on the utilization of each part of MCOC, there was scarce evidence on risk factors of dropout from institutional delivery among ANC booked HIV-positive mothers. Thus, this study aimed to assess risk factors of dropout from institutional delivery among ANC booked HIV-positive mothers under care in Gondar city public health facilities within one year postpartum.

## Methods

### Study design, period, and settings

A multicenter case–control study was conducted from May 01 to June 30/2018. The study was undertaken in Gondar city’s public health institutions. Gondar city is located 750 km away from Addis Ababa-the capital city of Ethiopia. According to the 2018/19 population projection, the total population size of the city is estimated to be 338,646, of whom, about 23.58% were women in the reproductive age group [18]. In Gondar city, there are nine public health facilities: one comprehensive specialized hospital and eight public health centers. In addition, there are three private maternity specialty clinics and one private primary hospital. All health facilities are currently providing maternal health care services.

### Eligibility criteria

We included ANC-booked HIV-positive women under care at Antiretroviral Therapy (ART) clinics in public health institutions in Gondar city within one year postpartum. Cases and controls were assigned based on the respondents’ place of delivery for their youngest child. Accordingly, ANC-booked HIV-positive women who underwent home delivery have been assigned to the case group, whereas, those ANC-booked HIV-positive women who gave birth at the health facility were enrolled in the control group.

### Sample size determination and sampling technique

The sample size for the study was determined using Open Epi version 3 software, by considering the following parameters; level of significance – 95%, power – 80%, ratio of cases to controls – 1: 3, the proportion of controls with ≤ 3 number of ANC – 29.25%, Odds Ratio (OR) – 2.55, percent of cases with ≤ 3 number of ANC visits – 52.56% from a previous similar study done in Southern Ethiopia [8]. Thus, the minimum adequate sample size for this study was obtained to be 202. By considering a 10% non-response rate, the final sample size is turned to be 222 individuals (56 cases and 166 controls).

We included all women who fulfilled the case and control definition incomplete ascertainment fashion till the required sample size for cases and controls were obtained respectively.

### Variables of the study

The outcome variable for this study is a dropout from institutional delivery and which was dichotomized as “Case” and “Control”. Whereas, the explanatory variables included: socio-demographic variables such as the age of the mother, age of the child, sex of the child, religion, marital status, residence, mother’s educational status, spousal educational status, mother’s occupational status, spousal occupational status, Radio/TV and monthly income, and obstetric related variables including gravidity, parity, time of ANC initiation, number of ANC visit, duration of rupture of membrane (ROM), obstetric complication during pregnancy, duration of labor, duration of ART drug initiation and provision of counseling about PMTCT at the health facility.

### Operational or term definitions

#### ART Clinics

Stand for both ART and PMTCT clinics.

#### ANC booked women

Those HIV-positive women who have received at least one antenatal care and registered from at least one of the public health facilities in Gondar city during the most recent pregnancy.

#### Dropout from institutional delivery

ANC booked women who delivered the indexed child at home.

#### Case

ANC booked HIV-positive women giving birth at home within one year before the data collection date and attending ART clinic during the data collection period.

#### Control

ANC booked HIV-positive women giving birth at health institutions within one year before the data collection date and attending ART clinics during the data collection period.

### Data collection tools, methods, and procedures

Data were collected by using pretested and structured questionnaires through a face-to-face interview. In addition, a checklist was used to extract certain variables via a medical chart review. Ten unemployed midwifery graduates (i.e., 8 diploma midwives for data collection and 2 BSc midwives for supervision) were recruited for the data collection process. A one-day training was provided. Pretest was done on 5% of the sample size. The necessary revision was then made on the tools after the pretest for further clarity. Daily supervision has been undertaken and the respective feedbacks have been provided. The questionnaire was adapted from different previous related literature [8, 19–21] and prepared first in English then translated into Amharic (local language), and finally back to English to maintain the consistency of the tool. Each questionnaire was reviewed daily for completeness and clarity. Furthermore, the data collectors have checked the questionnaire for completeness ahead of leaving the respondents.

### Data processing and analysis

We checked, coded, and entered the Data into EPI INFO version 7. Then, we exported the data to SPSS version 25 for analysis. We processed the data for both descriptive and analytical statistics. Descriptive statistics such as frequency, percent, and median were yielded and reported in texts and tables. Analytical statistical procedures were made via a Binary logistic regression model to identify risk factors of dropout from institutional delivery. Both bivariable and multivariable logistic regression analyses were performed. Both COR and AOR with the corresponding 95% CI were computed. Finally, the level of significance was decided based on AOR with its 95% CI at a p-value of ≤ 0.05.

## Result

### Socio-demographic characteristics of the participants

A total of 222 HIV-positive mothers were included in the study, yielding a response rate of 100%. About 26 (46.42%) of the cases and 50 (30.12%) of the controls gave the most recent birth at the age of ≥ 35-year-old with the median age of 32 years (IQR = 28–40) and 31 years (IQR = 28–35) respectively. The majority of the study participants (i.e., 89.29% of the cases and 92.17% of the controls) were urban residents. Large proportions of the respondents (57.14% of the cases and 80.12% of the controls) were Orthodox Tewahido religion followers. About 28 (50%) of the cases and 35(21.08%) of the controls were single at the time of the interview. More than two-fifth (44.64%) of the cases and one-third (34.34%) of the controls had never attended any formal education. Similarly, about half (i.e., 50% in the case group and 50.6% in the control group) of the participants were housewives by occupation. A large proportion (82.14%) of the respondents in the case group and considerable (46.39%) of the respondents in the control group had earned ≤ 36 US dollars monthly with a median family income of 19.64 US dollars (IQR = 11.61–35.71) and 53.57 (IQR = 21.43–107.14) respectively [See Table 1].

**Table 1:** Sociodemographic characteristics of the respondents under care in Gondar city’s governmental health institutions, Northwest Ethiopia (*n* = 222)

Variables	Groups for Dropout	
	Cases	Control
**Age of the mother** (in year)		
≤ 35	30	116
≥ 35	26	50
**Sex of child**		
Male	24	80
Female	32	86
**Residence**		
Rural	6	13
Urban	50	153
**Monthly income (US dollar)**		
≤ 36.00	46	77
> 36.00	10	89
**Radio /TV**		
Yes	49	129
No	7	37
**Religious**		
Orthodox Tewahido	32	133
Muslim	24	32
**Marital status**		
Currently single	28	35
Currently married	28	131
**Mother educational status**		
Unable to read and write	21	45
Able to read and write	35	121
**Spousal educational status**		
Unable to read and write	11	19
Able to read and write	45	147
**Mother occupation**		
Housewife	28	84
Unemployed	23	51
Governmental employed	5	31
**Spousal occupation**		
Employed	9	68
Unemployed	47	98
**Age of the child (months)**		
≤ 2	14	85
2–6	12	30
> 6	9	25

### Obstetric related characteristics of the participants

Primigravida women constituted nearly one-sixth (16.07%) in the case group and one-fourth (21.08%) in the control group with the median gravidity number of 3 (IQR = 2–4) and 2 (IQR = 2–3) for the cases and controls respectively. About 20 (35.71%) of the cases and 40 (24.10%) of the controls received incomplete ANC in the recent pregnancy with a median number of ANC visits of 4 (IQR = 3–4) and 4 (4–4) respectively. Whereas, nearly one-third (32.14%) of the cases and one-fourth (23.49%) of the controls initiated their ANC visit lately. About 2 (3.57%) among the cases and 43 (25.90%) among the controls had experienced obstetric complications during the index pregnancy. All women in both case and control groups admitted that they have been counseled on PMTCT during ANC follow-up. A considerable proportion (25%) of the cases and a smaller proportion (3.01%) of the controls had practiced non-recommended infant feedings [See Table 2].

**Table 2:** Obstetric related characteristics of the respondents under care in Gondar city’s governmental health institutions, Northwest Ethiopia (*n* = 222)

Variables	Groups for Dropout	
	Cases	Control
**Number of ANC**		
≤ 3	20	40
≥ 4	36	126
**ANC initiation (weeks)**		
≤ 16	38	127
> 16	18	39
**Gravidity**		
Primigravida	9	35
Multigravida	47	131
**Parity**		
≤ 3	40	143
≥ 4	16	23
**Obstetric complication**		
Yes	2	43
No	54	123
**Infant feeding practice**		
Recommended	42	161
Non-recommended	14	5
**Duration of labor (hrs)**		
≤ 12	41	134
> 12	15	32
**Duration of ART (year)**		
< 4	28	53
≥ 4	28	113
**Duration of ROM (hrs.)**		
< 4	40	151
≥ 4	16	15

### Risk factors of dropout from institutional delivery among study participants

We carried out both bivariable and multivariable logistic regression analyses. Based on the bivariable analysis, maternal age of ≥ 35 years, single marital status, unemployed spousal occupation, family monthly income of ≤ 36 US dollars, parity of ≥ 4, and no obstetric complication during the recent pregnancy were significantly associated with dropout from institutional delivery. In the multivariable logistic regression analysis, however, age of ≥ 35 years, currently single marital status, unemployed spousal occupation, family monthly income of ≤ 36 US dollars, and no obstetric complication during the recent pregnancy had a statistically significant association with dropout from institutional delivery.

Accordingly, mothers getting pregnant at advanced age were 2.37 times more likely to commit dropout from institutional delivery compared with the reference group (AOR = 2.37; 95% CI: 1.13,5.13). Similarly, single women were 3.28 times more probably to undergo home delivery as compared to married women (AOR = 3.28; 95% CI: 1.51,7.13). Women having unemployed spouses were more likely to experience dropout from institutional delivery services compared with those women having an employed spouse (AOR = 3.91; 95% CI: 1.54, 9.91). Similarly, the odds of dropout from institutional delivery were 4.87 times higher among women whose families earned ≤ 36 US dollars monthly than those women with > 36.00 US Dollars (AOR = 4.87; 95% CI: 2.08,11.42). Finally, women with no obstetric complications in the most recent pregnancy were 13.89 folds more probably to get dropout as compared to those women who had complications (AOR = 13.89; 95% CI: 2.73,27.71) [See Table3].

**Table 3:** Risk factors of dropout from institutional delivery among ANC booked HIV positive mothers, Northwest Ethiopia (*n* = 222)

Variables	Groups for Dropout		COR with 95% CI	AOR with 95% CI
	Cases	Controls		
**Age of the mother** (in year)				
< 35	30	116	1	1
≥ 35	26	50	2.01(1.10,3.74)	**2.37(1.13,5.13)**
**Residence**				
Rural	6	13	1.41(0.51,3.91)	0.58(0.16,2.04)
Urban	50	153	1	1
**Marital status**				
Currently single	28	35	3.74(1.97,7.12)	**3.28 (1.51,7.13)**
Currently married	28	131	1	1
**Mother educational status**				
Unable to read and write	21	45	0.52(0.23,1.17)	1.10(0.42,2.88)
Able to read and write	35	121	1	1
**Spousal educational status**				
Unable to read and write	11	19	1.89(0.84,4.27)	1.00(0.32,3.13)
Able to read and write	45	147	1	1
**Mother occupation**				
Housewife	28	84	2.07(0.73,5.83)	0.56(0.14,2.17)
Unemployed	23	51	2.80(0.96,8.11)	0.28(0.06,1.33)
Governmental employed	5	31	1	1
**Spousal occupation**				
Employed	9	68	1	1
Unemployed	47	98	3.62(1.67,7.88)	**3.91(1.54,9.91)**
**Monthly income(US dollar)**				
≤ 36.00	46	77	5.32(2.52,11.24)^a^	**4.87(2.08,11.42)**^**a**^
> 36.00	10	89	1	**1**
**Radio /TV**				
Yes	49	129	2.01(0.84,4.80)	1.79 (0.57,2.92)
No	7	37	1	1
**Number of ANC**				
≤ 3	20	40	0.57(0.30,1.10)	1.65(0.74,3.69)
≥ 4	36	126	1	1
**ANC initiation (in weeks)**				
≤ 16	38	127	1.54(0.79,3.00)	1.84(0.47,7.20)
> 16	18	39	1	1
**Gravidity**				
Primigravida	9	35	0.72(0.32,1.60)	0.57(0.19,1.67)
Multigravida	47	131	1	1
**Parity**				
≤ 3	40	143	1	1
≥ 4	16	23	2.49(1.20,5.15)^a^	1.86(0.68,5.05)
**Obstetric complication**				
Yes	2	43	1	1
No	54	123	9.44(2.21,40.38)^a^	**13.89 (2.73, 27.71)**^**a**^

^a^*p* < 0.001

## Discussion

In Ethiopia, under the current SDG period, the welfare of mothers, newborns and children remains a top priority for the health sector, but a significant proportion of women dropped out of maternity healthcare services utilization after booking for ANC. Recent nationwide evidence revealed that more than one in four (26%) of the ANC booked postnatal women failed to receive facility-based delivery services [22]. Moreover, only 6.58% of postnatal women utilized all the recommended MCOC [23]. This figure implies that there was a widespread missed opportunity of building rapport, fostering client-provider partnership, and gaining a positive first impression at the very beginning of ANC contact. These could result in dissatisfaction with ANC which is a prominent barrier to clients’ retention in the continuum of maternity care services. These missed opportunities and dissatisfaction with ANC could bring about low MCOC utilization and poor pregnancy outcomes for the general population [24]. On top of that, poor adherence to the uptake of maternity services among HIV-positive women worsens the problems as it is consistently associated with vertical transmission of HIV from mother to child and indirect cause of maternal mortalities [25, 26].

Therefore, effective interventions that could improve MCOC utilization for the general population and HIV-positive women, in particular, are imperative. In this perspective, identifying determinants of dropout of MCOC is helpful to design and execute evidence-based interventions. Having this insight in mind, we conducted a case–control study to identify the risk factors of dropout from institutional delivery among ANC booked HIV-positive mothers. To the best of our knowledge, this study is the first of its kind to exclusively identify the risk factors of dropout from institutional delivery in the context of HIV in Ethiopia.

The key findings of this study point out the role of social determinants of health in maternity services in Ethiopia and the necessity of conducting further research using a triangulated methodological approach to address deep-rooted social determinants of health in the context of HIV. The findings also underpin the need for implementation and fidelity research for determining which strategies may be most effective in improving maternity health service utilization and health outcomes in the context of HIV in the country.

World Health Organization (WHO) defines social determinants of health as the conditions in which people are born, grow, work, live, and age and the wider set of forces and systems shaping the conditions of daily life [27]. Social determinants include factors such as socioeconomic status, education, employment, social support network, neighboring and physical environment, as well as access to health care [28]. Numerous social determinants of health play a much bigger role in influencing a person’s health than medical care itself. Evidence documented that social determinants accounted for 80–90% while medical care itself constituted only for 10–20% of the contributors to people’s health outcomes [29, 30]. Thus, addressing social determinants of health is not only important for improving overall health, but also for reducing health disparities that are deep-rooted in social and economic disadvantages.

This study identified factors like age ≥ 35-year-old, currently single marital status, unemployed spousal occupation, and lower family monthly income were identified as predictors of dropouts from institutional delivery among ANC booked HIV-positive mothers. In addition, the odds of home delivery were significantly higher among women with no obstetric complications in the index pregnancy than the counter groups.

The current study showed that maternal age was found to be an important predictor in affecting the dropout from institutional delivery among ANC booked HIV-positive mothers. It revealed that older HIV-positive women have a high probability of dropout from institutional delivery compared to younger women. The odds of dropout from institutional delivery was 2.37 times higher among HIV-positive mothers who belong to the age class of 35 years and above compared to those whose age is below 35 years. This could be ascribed because older women consider themselves as having experienced so they don’t need assistance from skilled workers. At the same time, this evidence may be linked to the chronification process of the HIV population because as age increases there are patients who suffer from other conditions [31]. Further, discrimination and rejection guide people’s lives, associating with stigma and prolonged severe psychological trauma which may influence birth decisions [10]. Thus, stigma and discrimination are strong parameters in creating hidden humanity that is extremely difficult to reach, which is mainly seen as a syndrome of older age [32]. The finding is concise with those of different studies conducted so far in Zambia [33], Tanzania [34], Kenya [35], and Nepal [36].

In this study, single HIV-positive women were 3.28 times more likely to dropout from institutional delivery and prefer to give birth at home compared with married ones. Consistent with other study findings conducted in Nigeria [9], Zambia [33], and Malawi [20]. This could be clarified by the reason that numerous pregnancies among single HIV-positive women likely tend to be stigmatized, for unintended pregnancies, ANC follow-up is unlikely and family pressure may keep them at home since pregnancy before marriage is disgraceful for the family. Also married HIV-positive women, as estimated by plentiful health outcomes are commonly healthier than unmarried individuals [37, 38]. The significance of emotional support is evident in studies that show a strong correlation between lack of support in unmarried people even with normal mental status [32, 39].

We found an interesting association between a husband’s occupation and dropout from institutional delivery. Thus, a higher proportion of HIV-positive mothers whose spouses were farmers, daily laborers, and merchants by occupation other than those respondents whose spouses were employed in either governmental or private organizations dropped out from the institutional delivery. Similarly, preference for home birth was more observed in the mothers whose husband’s occupation was not office worker [40]. This result could be due to people working in governmental and private organizations usually being educated and might have a better chance to access information and use health services when compared to unemployed ones [41]. Moreover, it might be because an employed person would have control of their economy and therefore be more likely to use the healthcare services as compared with those who were unemployed [42]. Then, financial autonomy is insufficient to assert women’s empowerment.

Furthermore, monthly income was another risk factor that positively affects the occurrence of dropout from institutional delivery among HIV-positive mothers. Accordingly, the likelihood of dropout from institutional delivery among HIV-positive mothers who earned ≤ 36 US Dollars was more likely to dropout from institutional delivery compared to those who earned > 36 US dollars. This finding is congruent with other local studies in Ethiopia [41, 43, 44]. This might be explained by the fact that mothers who belong to poor families will most likely be unable to manage the cost of transportation and other backhanded expenses like medical costs. In Ethiopia, even though the service in a health post is given free of charge, it incurs costs when complicated delivery is referred to health centers. Also, mothers of low financial status may have lower access to health information. Low income is not a single factor but rather is characterized by multiple physical and psychosocial stressors. HIV status often hurts socioeconomic status by constraining an individual's ability to work and earn income, as well as poorer medication adherence and less favorable attitudes toward health care providers which in turn poor coverage of healthcare service utilization [23, 44, 45]. This finding suggests a vicious poverty-HIV/AIDS cycle in which HIV-infected people who are vulnerable to poverty are more likely to engage in high-risk behavior to cope with poverty. Therefore, the health impact of social factors also is supported by the strong and widely observed associations between a wide range of health indicators and measures of individuals’ socioeconomic resources or social position, typically income [29].

The current study also revealed that the odds of dropout from institutional delivery among HIV-positive mothers with no obstetric complications in the index pregnancy is higher than women who had an obstetric complication. This finding is consistent with previous local studies conducted in Ethiopia [8, 21, 46–48] and other studies carried out in Tanzania [19], Kenya [10], and Nepal [36]. The evidence for an association between HIV and other direct obstetric complications was inconsistent. Whilst HIV was associated with an increased risk of antepartum hemorrhage, there was no evidence of an increased risk of placenta praevia, placental abruption, postpartum hemorrhage, or retained placenta [49]. However, this association could be linked to the fact that women’s rich experiences with such complications could help them to visit health facilities thereby gaining a comprehensive and favorable awareness of maternal healthcare services [24]. Thus, women will build up a feeling of subsequent susceptibility to serious childbirth complications and are therefore more likely to look for help from health professionals.

### Limitations of the study

The study includes HIV-positive antenatal care booked mothers who gave birth outside health institutions. Therefore, the sensitivity of the topic by itself social desirability bias may be introduced, which can lead to underestimating the socio-cultural and behavioral determinants. The study also might have measurement error as our binary classifications of some variables (i.e. age, education status, marital status).

## Conclusion

The risk factors of dropout from institutional delivery in the context of HIV were connected to social determinants of health such as advanced maternal age, unmarried marital status, unemployed husband occupation, and low family income. Therefore, interacting with the health system by focusing on these women in lower socio-economic strata and unmarried HIV-positive ANC attendees and increasing access to information on obstetric complications during the ANC visit is important for retention of clients in the MCOC. Further innovative advocacy programs that help to bridge the gap between ANC attendance and institutional delivery are highly recommended to improve MCOC in Ethiopian women. There is a need for a more holistic prevention framework regarding dropout from a continuum of care targeting the aforementioned social determinants of health.

## Supplementary Information

**Additional file 1.**

## Data Availability

The datasets employed in the current study can be available from the corresponding author upon reasonable request.

## Abbreviations

ANC: Antenatal Care
ART: Antiretroviral Therapy
MCOC: Maternity Continuum of Care
MTCT: Mother to Child Transmission
PMTCT: Prevention of Mother to Child Transmission
ROM: Rupture of Membrane
SBA: Skilled Birth Attendant
SD: Standard Deviation
SSA: Sub-Saharan Africa
